# Assessing Additive Interactions between Protective Factors Using Relative Risk Reduction Due to Interaction

**DOI:** 10.3390/medicina60071053

**Published:** 2024-06-26

**Authors:** Andrea Nova, Teresa Fazia, Luisa Bernardinelli

**Affiliations:** Department of Brain and Behavioral Sciences, University of Pavia, 27100 Pavia, Italy; teresa.fazia01@ateneopv.it (T.F.); luisa.bernardinelli@unipv.it (L.B.)

**Keywords:** additive interaction, synergism, protective factors, moderation, effect modification

## Abstract

*Background and Objectives*: In the context of disease prevention, interaction on an additive scale is commonly assessed to determine synergistic effects between exposures. While the “Relative Excess Risk due to Interaction” represents the main measure of additive interaction between risk factors, in this study we aimed to extend this approach to assess additive interaction between factors known to prevent the event’s occurrence, such as medical interventions and drugs. *Materials and Methods*: We introduced and described the “Relative Risk Reduction due to Interaction” (RRRI) as a key measure to assess additive interactions between preventive factors, such as therapeutic interventions and drug combinations. For RRRI values closer to 1, the combination of exposures has a greater impact on reducing the event risk due to their interaction. As a purely illustrative example, we re-evaluated a previous investigation of the synergistic effect between statins and blood pressure-lowering drugs in preventing major adverse cardiovascular events (MACE). Moreover, simulation studies were used to empirically evaluate the performance of a robust Poisson regression model to estimate RRRI across different scenarios. *Results*: In our example, the drug combination revealed a positive additive interaction in further reducing MACE risk (RRRI > 0), even if not statistically significant. This result is more straightforward to interpret as compared to the original one based on the RERI. Additionally, our simulations highlighted the importance of large sample sizes for detecting significant interaction effects. *Conclusion*: We recommend RRRI as the main measure to be considered when exploring additive interaction effects between protective exposures, such as the investigation of synergistic effects between drug combinations or preventive treatments.

## 1. Introduction

In the public health context, a common research question is used to pinpoint specific subgroups within the population where interventions or treatments are expected to yield the most significant impact. In other words, it is of interest to assess if there is an interaction between two factors, implying that these are not acting independently to influence a specific outcome [1]. The presence of an interaction is commonly assessed using statistical models evaluating the deviation from additivity or multiplicativity. On an additive scale, interaction means that the combined effects of two factors is either greater or lesser than the sum of their individual effects [2]. Instead, on a multiplicative scale, interaction implies that the combined effect is either greater or lesser than the product of their individual effects. Therefore, assessing statistical additive interactions between exposures has a different meaning compared to evaluating statistical multiplicative interactions or evaluating risk ratios for one exposure within the strata of the other exposure. While guidelines suggest reporting interaction using both scales [2,3], evaluating interactions on an additive scale is considered more relevant in the context of disease prevention, as it is more suitable to determine how best to allocate medical interventions and treatments [4]. The reader can find practical examples and thorough explanations in [3]. Moreover, an additive interaction more closely corresponds to a mechanistic interaction, as it suggests that there are certain individuals for whom the outcome occurs only when both exposures are present [5]. Current literature describes measures of interaction on an additive scale, such as the relative excess risk due to interaction (RERI), which was originally developed for risk factors rather than protective factors [6]. It was proposed that when dealing with protective factors, one should transform them into risk factors before applying these measures [7]. This transformation involves selecting the reference category as the stratum with the lowest risk. However, in cases where both factors are known to be protective, such as in the case of therapeutic interventions with drugs or gene–environment interactions between preventive exposures and protective genetic polymorphisms [8], we show here that additive interactions can be assessed more appropriately without changing the reference category. In this context, we introduce and describe the Relative Risk Reduction due to Interaction (RRRI). This measure represents a proper measure to assess additive interaction for protective factors, as it considers risk reduction rather than excess risk. This measure equivalently assesses the presence additive interaction at the risk scale and the survival probability scale, i.e., probability of not having the event. Using a real-data scenario, we show as a purely illustrative example a potential practical application of the RRRI in the context of drug combinations, underlining the higher interpretability and suitability compared to the RERI when dealing with protective factors. Finally, to evaluate the efficiency of a robust Poisson regression model [9] to estimate RRRI, we conducted simulation studies to assess the model’s performance in terms of bias, statistical power, and coverage rate. We explored different scenarios varying sample size, proportion of exposed individuals, baseline risk, and RRRI magnitude.

## 2. Materials and Methods

### 2.1. Absolute Risk Reduction Due to Interaction

The standard method for evaluating interaction is to quantify how much the combined effect of both exposures on a binary event surpasses the impact of each exposure when considered separately. Rothman and Greenland’s formula [10] provides a means to assess the presence of additive interaction between two exposures calculating excess risks as follows:

(1) $$\left(R_{A+B+}-R_{A-B-}\right)={(R}_{A+B-}-R_{A-B-})+(R_{A-B+}-R_{A-B-})+AERI.$$

In this context, A and B denotes two different exposures, + and − denote, respectively, being or not being exposed, R represents the risk of event occurring within a particular subgroup (e.g., $A_{+}B_{+}$) and AERI denotes Absolute Excess Risk due to Interaction. In case of no additive interaction, AERI is equal to 0. From Equation (1), one can derive AERI as follows:

(2) $$AERI=R_{A+B+}-R_{A+B-}-R_{A-B+}+R_{A-B-}.$$

However, when examining protective factors, it is more straightforward to focus on risk reduction instead of excess risks. Multiplying both sides of Equation (1) for the term −1, one obtains the corresponding formula considering risk reductions:

(3) $$\left(R_{A-B-}-R_{A+B+}\right)={(R}_{A-B-}-R_{A+B-})+(R_{A-B-}-R_{A-B+})-AERI.$$

Therefore, the negative of AERI now denotes the Absolute Risk Reduction due to Interaction (ARRI):

(4) $$-AERI={-(R}_{A+B+}-R_{A+B-}-R_{A-B+}+R_{A-B-})=ARRI.$$

In this way, Equation (4) provides a means to assess the presence of additive interaction between two exposures which are known to reduce the event risk.

Equation (3) can also be rewritten in term of survival probabilities, denoted as S = 1−R, i.e., the probability of not having the event:

(5) $$\left(S_{A+B+}-S_{A-B-}\right)={(S}_{A+B-}-S_{A-B-})+(S_{A-B+}-S_{A-B-})+AESI$$

where AESI corresponds to the Absolute Excess Survival due to Interaction, which is also equal to the ARRI:

(6) $$AESI=S_{A+B+}-S_{A+B-}-S_{A-B+}+S_{A-B-}=ARRI.$$

Therefore, there is a one-to-one correspondence for interaction on an additive scale for both risks and survival probabilities, and one can describe both AESI and ARRI equivalently in terms of excess of absolute survival probability or absolute risk reduction. 

### 2.2. Relative Risk Reduction Due to Interaction

Next, we show how to assess an additive interaction when Relative Risks (RRs) are available, rather than absolute risks. By dividing all risks in Equation (3) by the baseline risk in the non-treated subgroup, $R_{A-B-}$, additive interactions can be evaluated calculating relative risk reductions, i.e., subtracting the RRs from 1.

(7) $$\left(1-{RR}_{A+B+}\right)=\left(1-{RR}_{A+B-}\right)+\left(1-{RR}_{A-B+}\right)+RRRI$$

where RRRI denotes the Relative Risk Reduction due to Interaction:

(8) $$\begin{array}{c} RRRI=\frac{ARRI}{R_{A-B-}}=\left(1-{RR}_{A+B+}\right)-\left(1-{RR}_{A+B-}\right)-(1-{RR}_{A-B+})\\ =-{(RR}_{A+B+}-{RR}_{A+B-}-{RR}_{A-B+}+1) \end{array}$$

As one can notice, RRRI corresponds to the negative of RERI. However, since relative risk reductions are restricted between 0 and 1, RRRI can range from −2 (perfect sub-additivity) to 1 (perfect super-additivity). RRRI=1 implies that the absolute baseline risk, $R_{A-B-},$ is completely removed due to the additive interaction between the individual effects. 

We will now show that Equation (8) can be generalized when dealing with Odds Ratios (ORs) from case-control studies, and with Hazard Ratios (HRs) in proportional hazard models from prospective cohort studies.

### 2.3. Obtaining RRRI When Dealing with Odds Ratios

The previously illustrated approach to evaluate additive interaction using protective factors can also be employed when dealing with case-control data, given that the ORs closely approximate the RRs (such as when the event is rare [11]). RRRI can be then obtained using Equation (8) considering ORs instead of RRs:

(9) $$\begin{array}{c} RRRI=\left(1-{OR}_{A+B+}\right)-\left(1-{OR}_{A+B-}\right)-(1-{OR}_{A-B+})\\ =-{(OR}_{A+B+}-{OR}_{A+B-}-{OR}_{A-B+}+1). \end{array}$$

### 2.4. Obtaining RRRI When Dealing with Hazard Ratios

The approach to obtain RRRI is also valid for proportional hazards models. Following the work in [12], considering hazard rates λ at a given time t, additive interactions can be assessed using the formula:

(10) $$\left(\lambda_{A+B+}-\lambda_{A-B-}\right)={(\lambda }_{A+B-}-\lambda_{A-B-})+(\lambda_{A-B+}-\lambda_{A-B-})+AERI$$

where AERI is defined as:

(11) $$AERI=\lambda_{A+B+}-\lambda_{A+B-}-\lambda_{A-B+}+\lambda_{A-B-}.$$

As previously described, when dealing with protective factors, it is more appropriate to focus on the absolute risk reduction. Therefore, the previous formula can be rewritten considering absolute risk reductions as:

(12) $$\left(\lambda_{A-B-}-\lambda_{A+B+}\right)={(\lambda }_{A-B-}-\lambda_{A+B-})+(\lambda_{A-B-}-\lambda_{A-B+})-AERI.$$

As in Equation (4), the negative of AERI now denotes the ARRI. Thus, for a given time t, additive interaction for protective factors can be then assessed using the formula:

(13) $$-AERI={-\lambda }_{A+B+}+\lambda_{A+B-}+\lambda_{A-B+}-\lambda_{A-B-}=ARRI.$$

By dividing all hazard rates in Equation (12) by baseline hazard rate, $\lambda_{A-B-}(t)$, an equivalent expression to Equation (7) for HRs is obtained:

(14) $$\left(1-{HR}_{A+B+}\right)=\left(1-{HR}_{A+B-}\right)+\left(1-{HR}_{A-B+}\right)-RRRI.$$

Consequently, RRRI can be obtained as:

(15) $$RRRI=-{(HR}_{A+B+}-{HR}_{A+B-}-{HR}_{A-B+}+1).$$

RRRI > 0 implies that there is some time t>0 such that there is an additive interaction between A and B. More details can be found in [13].

Conversely to what is observed for risks, additive interaction at the hazard rate scale does not correspond to an additive interaction at the survival scale. The survival function is defined as:

(16) $$S\left(t\right)={exp}^{-\lambda t}=P(T>t)$$

where T denotes the time to event. Applying this transformation to Equation (12) one obtains:

(17) $${exp}^{\left(\lambda_{A-B-}-\lambda_{A+B+}\right)}={exp}^{{(\lambda }_{A-B-}-\lambda_{A+B-})}*{exp}^{\left(\lambda_{A-B-}-\lambda_{A-B+}\right)}*{exp}^{ARRI}.$$

Which leads to:

(18) $${RS}_{A+B+}={RS}_{A+B-}*{RS}_{A-B+}*{exp}^{ARRI}.$$

Consequently, ${exp}^{ARRI}=\frac{{RS}_{A+B+}}{{RS}_{A+B-}*{RS}_{A-B+}}$, represents the multiplicative interaction between RSs. The additive interaction at the hazard scale then corresponds to the multiplicative interaction at the survival scale.

### 2.5. Sufficient Cause Interaction

As illustrated in [2,14,15], assessing additive interaction can be used to obtain insights on the presence of interaction in a mechanistic sense other than from a purely statistical point of view. In this context, a sufficient cause interaction can be established between A and B, assuming that (i) the confounding between exposures A and B with the outcome is removed and that (ii) neither A or B can ever pose as a risk factor for any individual (negative monotonicity assumption). Denoting with $D_{AB}$ the counterfactual survival outcome, i.e., non-occurrence of the event, given that the individual was or was not exposed to treatments A and B, a sufficient cause interaction implies that there exists some individual such that $D_{A+B+}=1$ and $D_{A+B-}=D_{A-B+}=0$ [14]. Therefore, a sufficient cause interaction can be intended as a form of mechanistic interaction as it occurs when certain individuals experience the outcome only if both exposures are present, but not if only one of the exposures is present [2]. Provided that control is made for confounding of both exposures with the outcome and assuming that neither exposure can ever pose as a risk factor for any individual (negative monotonicity assumption), the following formula, as described in [16], implies the presence of sufficient cause interaction:

(19) $$\begin{array}{l} E\left(D|A=+,B=+\right)-E\left(D|A=-,B=+\right)-E\left(D|A=+,B=-\right)+\\ E\left(D|A=-,B=-\right)>0. \end{array}$$

Given that the previously mentioned assumptions are met, the Equation (19) can be evaluated for AESI>0, reflecting Equation (6), or, equivalently, ARRI>0 or RRRI >0.

### 2.6. Other Additive Interaction Measures

In addition to the RRRI, two other measures of interaction on an additive scale can be evaluated. The first is the attributable proportion of the joint effects of both exposures that is due to interaction (AP), which can be obtained equivalently considering both absolute and relative risks:

(20) $$AP=\frac{ARRI}{R_{A-B-}-R_{A+B+}}=\frac{RRRI}{1-{RR}_{A+B+}}.$$

AP can range from −∞ to 1. In the absence of additive interaction, AP=0. AP>0 implies super-additivity, while AP < 0 implies sub-additivity. 

The second measure is the ratio between the combined effect and the sum of the individual effects, called the synergy index (S), which can be obtained equivalently considering both absolute and relative risks:

(21) $$S=\frac{R_{A-B-}-R_{A+B+}}{{(R}_{A-B-}-R_{A+B-})+{(R}_{A-B-}-R_{A-B+})}=\frac{\left(1-{RR}_{A+B+}\right)}{\left(1-{RR}_{A+B-}\right)+(1-{RR}_{A-B+})}.$$

S ranges from 0 to ∞. In the absence of additive interaction, S=1. S>1 implies super-additivity, while *S* < 1 implies sub-additivity.

### 2.7. Inference

To test the null hypothesis RRRI=0, RRRI can be estimated using the parameters of a regression model. To estimate RRs, a logistic model can be implemented when the event is rare so that the odds approximate the risks. For more common outcomes, a robust Poisson regression model [2,9] (where the sandwich estimator is used to correct the variance for RR) or a log-binomial model [17] can be employed instead.

The model should include exposures A, B, and their product term A∗B to obtain the respective model parameters $\beta_{1}$, $\beta_{2},$ and $\beta_{3}$. RRRI can be obtained as:

(22) $${RRRI=-(exp}^{{(\beta }_{1}+\beta_{2}+\beta_{3})}-{exp}^{\beta_{1}}-{exp}^{\beta_{2}}+1).$$

Similarly, RRRI can be obtained also considering continuous exposures, following the approach illustrated in [2]:

(23) $${RRRI=-(exp}^{\left(A_{1}-A_{0}\right)*\beta_{1}+\left(B_{1}-B_{0}\right)*\beta_{2}+\left({A_{1}*B}_{1}-A_{0*}B_{0}\right)*\beta_{3}}-{exp}^{\left(A_{1}-A_{0}\right)*\beta_{1}}-{exp}^{\left(B_{1}-B_{0}\right)*\beta_{2}}+1).$$

where $A_{1}-A_{0}$ and $B_{1}-B_{0}$ denote the changes of interest for the expoures.

To obtain confidence intervals (CIs) for RRRI, bootstrap methods can be implemented. Alternatively, standard error for RRRI can be obtained using the delta method [18] or Zou’s “MOVER” approach [19]. If the aim of the study is to test the hypothesis of presence of additive interaction, evaluating the statistical power to detect a statistically significant RRRI estimate given the sample size is crucial, as larger sample sizes are needed to be able to detect significant interaction effects compared to main effects [2].

### 2.8. Simulation Studies

To empirically evaluate the performance of the proposed methods, we conducted simulation studies. The studies were performed in the context of a cohort design. For simplicity, we assumed the absence of any confounder variable. We simulated two binary protective exposures A and B as realizations of binomial distributions $X\sim B(N,p_{x})$, where N denotes the sample size and $p_{x}$ the probability of being exposed (assumed to be equal for both exposures). We simulated the binary outcome y for each individual as a realization of a binomial distribution, $y\sim B(N,p_{y})$, where $p_{y}$ denotes the probability of the outcome, equal to the inverse logit function of the linear predictor as follows:

(24) $$p_{y}=\frac{1}{1+exp(-\left(\beta_{0}+\beta_{1}X_{10}+\beta_{2}X_{01}+\beta_{3}X_{11}\right))}.$$

We then evaluated 72 different scenarios as the combination of the following parameters:

- Sample size N:5000,15,000,30,000.
- Probability $p_{x}$ of binary protective exposures A and B fixed to 0.20 and 0.50.
- Baseline risk in the non-exposed group $R_{A-B-}:0.05$ and 0.25. This is obtained fixing the intercept $\beta_{0}=\mathrm{logit}\left(R_{A-B-}\right)$.
- RRs for each combination and A and B, where the non-exposed group $A_{-}B_{-}$ represents the reference. In particular, as reported in Table 1, we fixed $\beta_{1}$, $\beta_{2}$, and $\beta_{3}$ such that the combinations of ${RR}_{A+B-}=\exp(\beta_{1})$, ${RR}_{A-B+}=exp(\beta_{2})$, and ${RR}_{A+B+}=exp(\beta_{3})$ lead to RRRI values equal to 0.101, 0.252, and 0.414.

For each of the 72 scenarios, 1000 replicates were performed. Using Equation (8), we estimated $\hat{RRRI}$ point estimates using the $\hat{RRs}$ obtained fitting a robust Poisson regression model [9]. In each replicate, we calculated the mean of all $\hat{RRRI}$, the mean bias from the true value and the Root Mean Square Error (RMSE), i.e., $RMSE=\sqrt{E[{\left(\hat{RRRI}-RRRI\right)}^{2}]}$. We fixed the I type error α = 0.05 and obtained a 95% CI using three different methods: assuming asymptotic normality, deriving the standard error using (i) the delta method [18] and (ii) Zou’s “MOVER” approach [19]; (iii) implementing the bootstrap bias-corrected and accelerated (BCa) method, fixing to 1000 the number of bootstrap replications with replacement. Within each scenario we evaluated (i) the statistical power, as the proportion of 95% CIs not containing the null value, i.e., 0, and (ii) the coverage rate as the proportion of 95% CIs containing the true value. To compare the extent to which the empirical coverage of the CIs matched with the nominal 95% level, we evaluated the proportion of coverage rates between 93% and 97% across all scenarios. Lastly, we conducted the same simulations fitting a log-binomial regression model to evaluate any potential differences in accuracy and precision (where delta method was used to estimate standard errors). 

Simulations were performed using RStudio 2024.04.1+748. In the Supplementary Materials we provided the R script which can be used to estimate and make inference on RRRI.

### 2.9. Example Dataset

To illustrate the explanation of the methods, we considered the data collected in [20], which aimed to investigate the combined effects of blood pressure-lowering drugs (BPRx) and statins in decreasing the risk of cardiovascular diseases. In that study, data were gathered from seven different randomized clinical trials for a total of 27,020 participants. For simplicity, we focus on Major Adverse Cardiovascular Events (MACE) as our outcome of interest. 

## 3. Results

### 3.1. Simulation Results

In Table S1, we report the results from the simulation studies after running 1000 replicates within each scenario using a robust Poisson regression model. Across all 72 scenarios, the coverage rate for RRRI 95% CIs resulted close to 1−α=95%, using both the bootstrap BCa method and assuming asymptotic normality. MOVER and delta method approaches gave similar performances in terms of coverage rates, which were between 93% and 97% in 68 scenarios (94%) using MOVER and in 67 scenarios (93%) using delta method. Statistical power was also similar, with MOVER leading to slightly higher power for $p_{x}=0.5$ while delta method for $p_{x}=0.2$. Generally, statistical power increases for higher N, RRRI, proportion of exposed individuals $p_{x}$, and $R_{A-B-}$. Similarly, RMSE decreases for higher N, $p_{x},$ and $R_{A-B-}$, while it does not appear to vary across different RRRI values. Given the same RRRI, a slight upwards bias appeared for combinations of lower RRs. Notably, N=30,000 was not sufficient to reach a statistical power >80% when the magnitude of additive interaction is low, i.e., RRRI=0.101. Instead, given RRRI=0.252, a statistical power >80% was reached for N=30,000 when baseline risk was low, i.e., $R_{A-B-}=0.05$, while N=15,000 was sufficient given $R_{A-B-}=0.25$. Finally, given RRRI=0.414, a statistical power >80% was achieved for N=15,000 when baseline risk was low, i.e., $R_{A-B-}=0.05$, while N=5000 was sufficient when $R_{A-B-}=0.25$. In Table S2, we reported the results from our same simulation studies using a a log-binomial regression model. The findings, in terms of bias, RMSE, statistical power, and coverage rate (using the delta method approach), are nearly identical to those obtained with the robust Poisson regression model. This indicates that both models are suitable for estimating and making inferences on RRRI.

### 3.2. Illustrative Example

In Table 2, the number of participants and MACE events, along with risks and RRs, is reported by stratum of treatment.

Considering absolute risks reductions, applying Equation (4) led to ARRI=−(0.041−0.033−0.039+0.027)=0.004 (which corresponds to the negative of AERI). ARRI>0 implies the presence of a positive additive interaction in reducing MACE risk, i.e., the combined effect of statins and BPRx in reducing MACE risk is larger than the sum of the individual treatment effects (super-additivity). Specifically, the absolute risk of MACE event is further reduced by 0.004 when both treatments are combined. Since ARRI=AESI, another alternative interpretation is that combining both treatments produces an absolute excess probability of not having MACE equal to 0.004. This would indicate that, given 1000 individuals, 4 additional MACE cases would be prevented if both treatments were used.

Considering RRs, applying Equation (8) leads to RRRI=−(0.642−0.932−0.801+1)=0.092. As for ARRI>0, RRRI>0 implies the presence of a positive additive interaction in reducing MACE risk (super-additivity). Specifically, when using both treatments, there is a further relative risk reduction equal to 0.092 compared to what is expected if there was no interaction between treatments.

To conduct statistical inference and test the null hypothesis RRRI=0, we implemented the bootstrap BCa method to obtain 5000 replications with replacements and derived a 95% CI for RRRI comprised between −0.116 and 0.340. We also obtained similar confidence limits deriving 95% CIs assuming asymptotic normality using the delta method and MOVER approaches to calculate standard errors, i.e., delta method 95%CI = −0.136,0.320 and MOVER 95% CI = −0.121,0.346. Since the null value is contained in the 95% CIs, we can conclude that there is not enough statistical evidence to support the hypothesis of an additive interaction between statins and BPRx to further decrease MACE risk. Finally, considering other measures of additive interactions, applying Equation (20) leads to AP=0.26, which implies that 26% of the risk reduction using both treatments is to be attributed to their joint effects (while the remaining 74% to the sum of their individual effects), while applying Equation (21) leads to S=1.34, which implies the presence of synergy between treatments. Both AP>0 and S>1 then correctly agree with RRRI>0, suggesting the presence of super-additivity.

## 4. Discussion

In this work, we described how to calculate measures of additive interaction when the exposures are known to be protective factors. In this scenario, there is no need to alter the way the variables are coded. Instead, it is more appropriate to consider risk reductions instead of excess risks. This consideration led to the formulation of the “Relative Risk Reduction due to Interaction” (RRRI), the formula of which corresponds to the negative of the “Relative Excess Risk due to Interaction” (RERI). RRRI serves as an indicator of how much the relative risk reduction is amplified when both exposures are present compared to the sum of the relative risk reductions when each factor is considered individually. Since risk reduction values fall within the range of 0 to 1, RRRI can assume values between −2 (indicating perfect sub-additivity) and 1 (indicating perfect super-additivity). Moreover, assessing additive interaction at the risk scale corresponds to assess additive interaction at the survival scale; therefore, a RRRI greater than 0 is equivalently linked to an excess survival probability of not experiencing the event due to the interaction. We also detailed how RRRI can be calculated in a similar manner using ORs, obtained from case-control studies, and HRs obtained from prospective cohort studies (if the respective assumptions are respected). In our illustrative example, we re-examined the study conducted in [20], which aimed to investigate synergistic effects between statins and BPRx in preventing MACE events. Their analysis found a RERI<0, which could be misinterpreted as a presence of negative interaction (sub-additivity) between the combined treatments in reducing MACE risk, i.e., there is an interaction between the combined treatments, which decreases the reduction in MACE risk. Instead, our calculation revealed a RRRI>0 (even though not reaching statistical significance), implying that the combined use of the two treatments exhibits a positive additive interaction (super-additivity) between the combined treatments, which further reduced MACE risk, i.e., there is an interaction between the combined treatments, which contribute to further reduce MACE risk. Finally, insights from our simulation studies offer valuable considerations when planning studies on additive interactions. First, we found a good performance in terms of CI coverage rate, assuming asymptotic normality [18,19]. Second, we further highlighted how large sample sizes, i.e., >5000 individuals, are necessary to detect significant interaction effects, especially when they are of small or modest magnitude, such as in the case of preventative medical treatments (e.g., drugs to prevent cardiovascular events) [2]. This sample size is not easily achievable using any design setting, especially when the aim is evaluating treatments in clinical randomized trials. However, as shown in our illustrated example, pooling information from several different studies could help to increase sample size and, consequently, the statistical power to detect significant additive interactions. Moreover, in scenarios where the outcome is common and a large effect size is expected, such as when evaluating progression-free survival for combined invasive therapies such as chemotherapy and surgery against cancer [21], a smaller sample size (e.g., ≈1000 to 5000 individuals) can provide sufficient statistical power. Lastly, it is also important to emphasize that even in studies with limited statistical power, estimating RRRI in an exploratory way can still yield valuable insights for guiding future research.

Among the limitations of the method, there is the potential for residual confounding between the exposures and the outcome, even after adjusting for known confounders [22]. Unknown or unmeasured confounders could still affect the observed associations, leading to biased estimates of causal additive interaction effects. To mitigate this issue, it is crucial to meticulously plan the study design and statistical analysis. Further research will focus on replicating this method using original data to better recognize the clinical implications.

## 5. Conclusions

In conclusion, we recommend using RRRI when exploring statistical interactions on an additive scale between two protective exposures aimed at preventing an event. This measure can be particularly valuable for assessing the synergistic effects of drug combinations or preventive treatments, as well as for gene–environment interactions between protective genetic polymorphisms and protective factors.

## Figures and Tables

**Table 1:** Relative Risks (*RRs*) for each combination of exposures A and B, leading to a different Relative Risk Reduction due Interaction (*RRRI*).

$\beta_{1}$	$\beta_{2}$	$\beta_{3}$	${RR}_{A+B-}$	${RR}_{A-B+}$	${RR}_{A-B+}$	RRRI
−0.100	−0.100	−0.345	0.905	0.905	0.708	0.101
−0.260	−0.260	−0.820	0.771	0.771	0.440	0.101
−0.120	−0.120	−0.650	0.887	0.887	0.522	0.252
−0.200	−0.200	−0.954	0.819	0.819	0.385	0.252
−0.150	−0.150	−1.180	0.861	0.861	0.307	0.414
−0.200	−0.200	−1.500	0.819	0.819	0.223	0.414

**Table 2:** Effects of statin treatment by subgroups of blood pressure-lowering treatment on Major Adverse Cardio-vascular Events.

	$\mathrm{No}\;\mathrm{BPRx}\;\mathrm{Use}\;(B_{-})$			$\mathrm{BPRx}\;\mathrm{Use}\;(B_{+})$		
	Events/N	Risk	RR (95% CI)	Events/N	Risk	RR (95% CI)
$\mathrm{No}\;\mathrm{Statins}\;\mathrm{use}\;(A_{-}$)	254/6125	0.041	1.000	204/6142	0.033	0.801 (0.668, 0.960)
$\mathrm{Statins}\;\mathrm{use}\;(A_{+}$)	236/6103	0.039	0.932 (0.784, 1.109)	163/6126	0.027	0.642 (0.529, 0.779)

RR = Relative Risk, BPRx = blood pressure-lowering drugs, N = sample size.

## Data Availability

No original data were used for this manuscript.

## Supplementary Materials

The following supporting information can be downloaded at: https://www.mdpi.com/article/10.3390/medicina60071053/s1, Table S1: Results from simulation studies over 1000 replicates within each scenario using a robust Poisson regression model. Table S2: Results from simulation studies over 1000 replicates within each scenario using a log-binomial regression model. R codes can be found in the script named “Code for RRRI estimation and inference.R”.
